# Natural History of Cutaneous Human Polyomavirus Infection in Healthy Individuals

**DOI:** 10.3389/fmicb.2021.740947

**Published:** 2021-10-18

**Authors:** Luisa Bopp, Ulrike Wieland, Martin Hellmich, Alexander Kreuter, Herbert Pfister, Steffi Silling

**Affiliations:** ^1^Institute of Virology, National Reference Center for Papilloma- and Polyomaviruses, University of Cologne, Faculty of Medicine and University Hospital of Cologne, Cologne, Germany; ^2^Department of Dermatology and Venereology, University of Cologne, Cologne, Germany; ^3^Institute of Medical Statistics and Computational Biology, Faculty of Medicine and University Hospital Cologne, University of Cologne, Cologne, Germany; ^4^Department of Dermatology, Venereology, and Allergology, Helios St. Elisabeth Hospital Oberhausen, University Witten-Herdecke, Witten, Germany

**Keywords:** polyomaviruses, skin, persistence, MCPyV, HPyV, TSPyV, STLPyV

## Abstract

Several human polyomaviruses (HPyVs) were recently discovered. Merkel cell polyomavirus (MCPyV) induces Merkel cell carcinoma. HPyV6, HPyV7, and TSPyV have been associated with rare skin lesions in immunosuppressed patients. HPyV9, HPyV10, and Saint Louis Polyomavirus (STLPyV) have not been convincingly associated with any disease. The aim of this prospective study was to evaluate the cutaneous prevalence, persistence and viral load of HPyVs in healthy individuals. Eight hundred seventy forehead and hand swabs were collected from 109 volunteers 4–6 weeks apart (collection period-1). Fifty-nine participants were available for follow-up a decade later (collection period-2). HPyV-DNA prevalence and viral loads of MCPyV, HPyV6, HPyV7, TSPyV, HPyV9, HPyV10, and STLPyV were determined by virus-specific real-time PCRs. Risk factors for HPyV prevalence, short- and long-term persistence were explored by logistic regression analyses. Baseline prevalence rates were similar for forehead and hand: MCPyV 67.9/67.0%, HPyV6 31.2/25.7%, HPyV7 13.8/11.0%, HPyV10 11.9/15.6%, STLPyV 7.3/8.3%, TSPyV 0.9/0.9%, and HPyV9 0.9/0.9%. Short-term persistence in period-1 was found in 59.6% (MCPyV), 23.9% (HPyV6), 10.1% (HPyV7), 6.4% (HPyV10), 5.5% (STLPyV), and 0% (TSPyV and HPyV9) on the forehead, with similar values for the hand. Long-term persistence for 9–12 years occurred only for MCPyV (forehead/hand 39.0%/44.1% of volunteers), HPyV6 (16.9%/11.9%), and HPyV7 (3.4%/5.1%). Individuals with short-term persistence had significantly higher viral loads at baseline compared to those with transient DNA-positivity (*p* < 0.001 for MCPyV, HPyV6, HPyV7, and HPyV10, respectively). This was also true for median viral loads in period-1 of MCPyV, HPyV6, and HPyV7 of volunteers with long-term persistence. Multiplicity (two or more different HPyVs) was a risk factor for prevalence and persistence for most HPyVs. Further risk factors were older age for HPyV6 and male sex for MCPyV on the forehead. Smoking was not a risk factor. In contrast to MCPyV, HPyV6, HPyV7, and rarely STLPyV, polyomaviruses TSPyV, HPyV9, and HPyV10 do not seem to be long-term constituents of the human skin virome of healthy individuals. Furthermore, this study showed that higher viral loads are associated with both short- and long-term persistence of HPyVs on the skin. HPyV multiplicity is a risk factor for prevalence, short-term and/or long-term persistence of MCPyV, HPyV6, HPyV7, and HPyV10.

## Introduction

Human polyomaviruses (HPyVs) are small, non-enveloped viruses with a double-stranded circular DNA genome. To date, 15 different HPyVs have been identified, with only some of them being associated with human disease, especially in immunosuppressed patients.

The first two HPyVs, BKPyV, and JCPyV, were discovered in 1971. Primary infection is usually asymptomatic, but reactivation is linked to serious diseases such as JCPyV-induced leukoencephalopathy or BKPyV-induced nephropathy in immunosuppressed patients (Gardner et al., 1971; Padgett et al., 1971). In 2007, KIPyV and WUPyV were discovered in respiratory secretions from patients with acute respiratory tract infections. However, their association with human disease is not well established (Allander et al., 2007; Gaynor et al., 2007). In 2008, Merkel cell polyomavirus (MCPyV) was described as an aetiological agent of Merkel cell carcinoma (MCC), a rare aggressive skin cancer of neuroectodermal origin (Feng et al., 2008). Since 2010, several novel HPyV species have been isolated. Four of them are possibly associated with rare skin lesions in immunosuppressed individuals: the skin-tropic HPyVs 6 and 7 were discovered on healthy human forehead skin in 2010 (Schowalter et al., 2010). Both of them are associated with pruritic and dyskeratotic dermatoses in immunosuppressed patients (Ho et al., 2015; Nguyen et al., 2017). Moreover, HPyV6 was detected in epithelial neoplasms such as keratoacanthomas (Schrama et al., 2014; Beckervordersandforth et al., 2016), while HPyV7 was found in thymomas (Rennspiess et al., 2015). However, their aetiological role in the development of these tumours remains unclear (Scola et al., 2012; Keijzers et al., 2015; Haeggblom et al., 2017; Amorrortu et al., 2021; Klufah et al., 2021). Trichodysplasia spinulosa–associated polyomavirus (TSPyV) was detected in immunosuppressed individuals with the rare skin disease trichodysplasia spinulosa, which is characterised by follicular keratotic papules and spicules in the face (van der Meijden et al., 2010; Kazem et al., 2013). New Jersey PyV (NJPyV-2013) was isolated from a muscle biopsy of a pancreatic transplant patient with vasculitic myopathy and retinal blindness in 2014 (Mishra et al., 2014). Five further HPyVs not associated with any disease have been identified in recent years (Ehlers and Wieland, 2013; Feltkamp et al., 2013; Moens et al., 2017): HPyV9 was discovered in the serum of a renal transplant patient in 2011 (Scuda et al., 2011). In 2012, HPyV10, or Malawi PyV (MWPyV), was detected coincidentally in perianal warts of a immunosuppressed patient as well as in stool samples of healthy children from Malawi (Buck et al., 2012; Siebrasse et al., 2012). Saint Louis PyV (STLPyV) was detected in stool specimens, and HPyV12 was found in liver, caecum, and rectum tissue (Korup et al., 2013; Lim et al., 2013) in 2013. In 2017, Gheit et al. (2017) reported the characterisation of a 14th HPyV, Lyon IARC PyV (LIPyV), from skin and oral gargle samples of healthy individuals. However, it cannot be excluded that HPyV12 and LIPyV may be contaminants from shrew and cat, respectively. Another probable HPyV, Quebec polyomavirus (QPyV), was recently identified in a metagenomic analysis of faecal samples of an elderly man and could also be detected by PCR in urine samples of immunosuppressed patients and pregnant women (Ondov et al., 2019; Prezioso et al., 2021).

Several studies have reported high seroprevalence (60–100%) for the majority of HPyVs in adults (Nicol et al., 2013; Van Der Meijden et al., 2013; Gossai et al., 2016; Kamminga et al., 2018). Lower seroprevalence rates (around 5%) were found for the recently identified HPyV12, NJPyV-2013, and LIPyV (Kamminga et al., 2018). Although serological evidence indicates that most adults have been immunologically exposed to the majority of HPyVs, the natural history of HPyV infections in healthy individuals has not yet been fully elucidated. To date, only a limited number of studies have assessed the prevalence of the recently detected HPyVs on the skin of healthy individuals, with the majority of them limited to men (Wieland et al., 2011, 2014; Hampras et al., 2015; Hashida et al., 2018). To identify individuals at high risk for infection and virus-associated disease, it is important to investigate the natural history of these potentially pathogenic viruses by studying their prevalence and persistence on human skin (Wieland et al., 2014; Hampras et al., 2015). The aim of this prospective study was to evaluate the cutaneous DNA prevalence, short- and long-term persistence and viral load of seven HPyVs with assumed skin tropism – MCPyV, HPyV6, HPyV7, TSPyV, HPyV9, HPyV10, and STLPyV – in healthy men and women.

## Materials and Methods

### Study Participants and Specimen Collection

**FIGURE 1:**
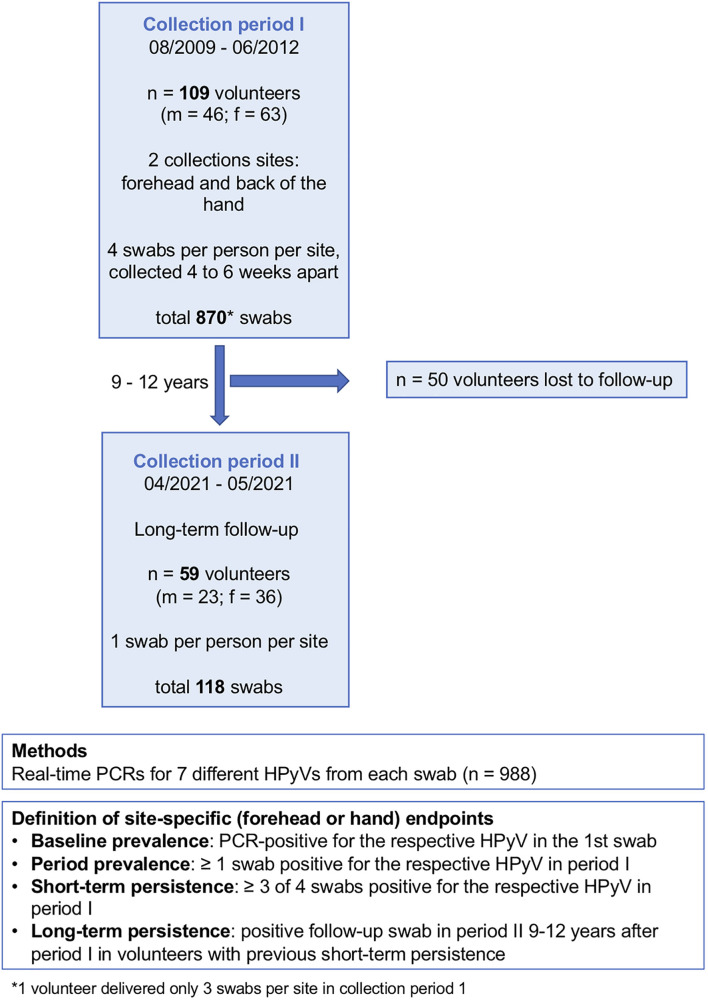
Study design. f, female; m, male; n, number; HPyV, human polyomavirus.

Within the first collection period (August 2009 to June 2012), 870 non-lesional skin swabs (forehead and back of the left hand) were collected from 109 healthy adult volunteers (Table 1 and Figure 1) at four points in time, each 4–6 weeks apart. For the long-term follow-up analysis, one forehead swab and one hand swab were taken between April and May 2021 (collection period-2) from the still-available volunteers (*n* = 59, 23 males, 36 females) 9–12 years after the initial collection period (Figure 1). Participants had to be free of skin cancer, skin infections, and acute or chronic inflammatory dermatoses. Data on the participants’ underlying diseases, immunosuppression and smoking status was collected. The study was approved by the ethics review board of the faculty of medicine of the University of Cologne (no. 10-230). Written informed consent was obtained from all participants. For sampling, a sterile cotton swab wetted with PBS was forcefully rubbed on the forehead including the eyebrows or on the back of the left hand of the participants.

**TABLE 1:** Study participants’ characteristics at baseline.

No. of participants		109
Sex – No. (%)	Female Male	63 (57.8) 46 (42.2)
Age – yr	Mean (range) Median (IQR)	41.6 (20–81) 39.0 (32.0–48.5)
Smoking status – No. (%)	Non-smoker Smoker	83 (76.1) 26 (23.9)
Acute or chronic dermatosis – No. (%)	No Yes	109 (100) 0
Chronic underlying disease* – No. (%)	No Yes	103 (94.5) 6 (5.5)
Immunosuppression – No. (%)	No Yes	108 (99.1) 1** (0.9)

*No., number; yr, year; IQR, interquartile range.*

*Diabetes mellitus (*n* = 3, males), Crohn’s disease (*n* = 1, female), cystic fibrosis (*n* = 1, female), rheumatoid arthritis (*n* = 1, female.

**Iatrogenic immunosuppression (sulfasalazine for rheumatoid arthritis).

### Human Polyomavirus DNA Detection

DNA isolation from the swab material was performed as previously described using a commercially available DNA extraction kit (QIAamp DNA Mini; Qiagen, Hilden, Germany) (Weissenborn et al., 2010; Wieland et al., 2011). Real-time human beta-globin-gene polymerase chain reaction (PCR) was performed to demonstrate that samples contained adequate DNA and were free of substances inhibitory to PCR [period-1: LightCycler Control Kit DNA; Roche, Mannheim, Germany; period-2: beta-Globin PCR according to the protocol of Van Duin et al. (2002)]. All samples were analysed by virus-specific quantitative real-time PCR (q-PCR) targeting the VP1 gene for the presence of MCPyV, HPyV6, HPyV7, TSPyV, HPyV9, and HPyV10 as previously reported (Weissenborn et al., 2010; Wieland et al., 2014). The same protocol was applied for the detection of STLPyV using primers 5′-GAAAATCTAGATGCACCTCACAGA-3′ and 5′-TTTGGCACGGATCATATTCA-3′ together with UPL-probe no. 63 (Roche, Mannheim, Germany, Cat no.: 04688627001). Viral loads were expressed as viral DNA-copies per beta-globin gene copy.

### Definitions

Baseline prevalence was defined as being positive for the respective HPyV in the first swab. Period prevalence was defined as being positive for the respective virus in ≥1 swab in period-1. Short-term persistence was defined as ≥3 of 4 swabs being positive for the respective HPyV in period-1. Long-term persistence was defined by a positive follow-up swab in period-2 in volunteers with previous short-term persistence (Figure 1).

### Data Analysis

Qualitative data were cross-tabulated and evaluated for association by the exact McNemar test, 2-sided, or the Fisher’s exact test, 2-sided. Distributions of HPyV DNA loads (forehead vs. hand) were tested for difference in location using the Wilcoxon signed-rank test. Distributions of HPyV DNA load (no persistence vs. persistence) were tested for difference in location using the non-parametric Mann–Whitney *U* test for two independent samples. The exact Clopper–Pearson approach was used for calculation of 95% confidence intervals (CI). Risk was modelled (explained) using univariable and multivariable logistic regression. Statistical analyses were done using Statistical Package for the Social Sciences (SPSS) Statistics 26 (IBM Corp., Armonk, NY, United States).

## Results

### Study Participants’ Characteristics

The participants’ characteristics at baseline are given in Table 1. 46 of the 109 volunteers were men, 63 were women. The age range was 20–81 years (mean age 41.6 years). Twenty-three point nine percent (*n* = 26) were smokers. At the time of sample collection, all participants were free of skin cancer, skin infections and acute or chronic inflammatory dermatoses. Six participants (5.5%) had a chronic underlying disease; one person was receiving immunosuppressive medication (see Table 1 for details).

### Site-Specific Human Polyomaviruses DNA Prevalence, Multiplicity, and Viral DNA Load at Baseline

At baseline, about three-quarters of the 109 participants were positive for any HPyV, 73.4% (95%CI 64.1–81.4) on the forehead and 80.7% (95%CI 72.1–87.7) on the back of the hand. Twenty-six point six percent (95%CI 18.6–35.9) of the volunteers did not carry any HPyV on the forehead, and in 19.3% (95%CI 12.3–27.9), no HPyV DNA was found on the hands at baseline. Baseline prevalence on the forehead and on the hand was highest for MCPyV (67.9%, 95%CI 58.3–76.5 and 67.0%, 95%CI 57.3–75.7, respectively), followed by HPyV6 (31.2%, 95%CI 22.7–40.8 and 25.7%, 95%CI 17.8–34.9). HPyV7, HPyV10, and STLPyV were less frequent and had similar baseline prevalence rates around 10% (see Table 2 for details). TSPyV and HPyV9 were detected in only 0.9% (95%CI 0–5.0) of baseline swabs, respectively (Table 2).

**TABLE 2:** Site-specific human polyomavirus (HPyV) prevalence, multiplicity, and viral load at baseline.

	Polyomavirus	Forehead	Hand	*p*-value^*,^°
**HPyV DNA prevalence** No. (%, 95%CI^#^) *n* = 109	MCPyV	74 (67.9, 58.3–76.5)	73 (67.0, 57.3–75.7)	1.000^*^
	HPyV6	34 (31.2, 22.7–40.8)	28 (25.7, 17.8–34.9)	0.263^*^
	HPyV7	15 (13.8, 7.9–21.7)	12 (11.0, 5.8–18.4)	0.581^*^
	TSPyV	1 (0.9, 0–5.0)	1 (0.9, 0–5.0)	1.000^*^
	HPyV9	1 (0.9, 0–5.0)	1 (0.9, 0–5.0)	1.000^*^
	HPyV10	13 (11.9, 6.5–19.5)	17 (15.6, 9.4–23.8)	0.289^*^
	STLPyV	8 (7.3, 3.2–14.0)	9 (8.3, 3.8–15.1)	1.000^*^
**Multiplicity** No. of different HPyVs per swab (%, 95%CI^#^) *n* = 109	All HPyVs negative	29 (26.6, 18.6–35.9)	21 (19.3, 12.3–27.9)	0.152^*^
	Any HPyV	80 (73.4, 64.1–81.4)	88 (80.7, 72.1–87.7)	0.152^*^
	Monoinfection	34 (31.2, 22.7–40.8)	54 (49.5, 39.8–59.3)	0.003^*^
	2 different HPyVs	29 (26.6, 18.6–35.9)	21 (19.3, 12.3–27.9)	0.215^*^
	3 different HPyVs	14 (12.8, 7.2–20.6)	7 (6.4, 2.6–12.8)	0.167^*^
	4 different HPyVs	3 (2.8, 0.6–7.8)	6 (5.5, 2.0–11.6)	0.250^*^
**Median viral DNA load** in HPyV-positive swabs (*n*, IQR)	MCPyV	0.37 (74, 0.06–5.00)	2.00 (73, 0.35–7.92)	**0.002**°
	HPyV6	0.06 (34, 0.01–0.29)	0.20 (28, 0.08–0.73)	0.683°
	HPyV7	0.05 (15, 0.01–0.73)	0.14 (12, 0.06–0.52)	0.478°
	TSPyV	0.002 (1)	0.42 (1)	0.655°
	HPyV9	1.43 (1)	0.38 (1)	0.317°
	HPyV10	0.04 (13, 0.02–0.05)	0.11 (17, 0.06–0.31)	**0.001**°
	STLPyV	0.01 (8, 0.01–0.04)	0.10 (9, 0.06–0.12)	**0.016**°

*No., number; CI, confidence interval; IQR, interquartile range.*

*^#^Exact Clopper–Pearson confidence intervals.*

*Exact McNemar test, two-sided. °Wilcoxon signed-rank test.

**p*-values that indicate statistically significant differences are printed in bold.*

Concomitant detection of more than one HPyV was found in 42.2% of baseline forehead swabs and in 31.2% of baseline hand swabs. In baseline forehead and hand swabs, two different HPyVs were detected in 26.6% (95%CI 18.6–35.9) and 19.3% (95%CI 12.3–27.9), three different HPyVs were detected in 12.8% (95%CI 7.2–20.6) and 6.4% (95%CI 2.6–12.8) and four different HPyVs in 2.8% (95%CI 0.6–7.8) and 5.5% (95%CI 2.0–11.6), respectively. Monoinfections occurred more frequently on the hand than on the forehead (*p* = 0.003; exact McNemar test, two-sided) (Table 2).

While there were no significant differences in site-specific HPyV detection rates, the median viral load for MCPyV, HPyV10, and STLPyV was significantly higher in hand swabs compared to forehead swabs (*p* = 0.002, *p* = 0.001, and *p* = 0.016, respectively, Wilcoxon signed-rank test) (Table 2).

**FIGURE 2:**
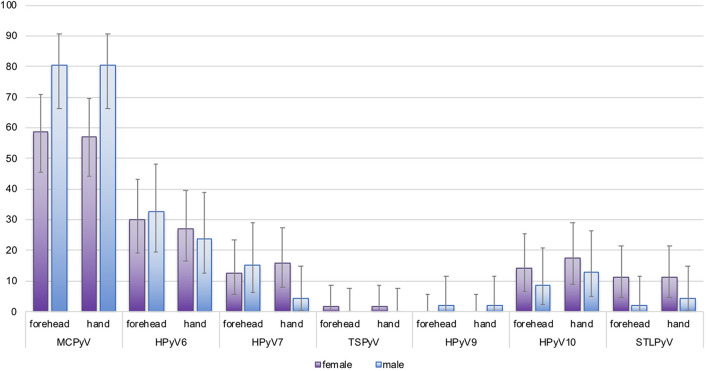
Site-specific human polyomavirus (HPyV) baseline prevalence by sex. Y-axis: prevalence in percentage (%) with error bars. Purple bars: females; blue bars: males. The difference in baseline prevalence between females and males was statistically significant for Merkel cell polyomavirus (MCPyV) (forehead, *p* = 0.022, hand *p* = 0.013; Fisher’s exact test, 2-sided).

MCPyV DNA prevalence at baseline was higher in forehead swabs of men (80.4%; 95%CI 66.1–90.6) compared to women (58.7%; 95%CI 45.6–71.0; *p* = 0.022). Similar results were observed for hand swabs (*p* = 0.013). For all other HPyVs, no significant differences in baseline detection rates in men compared to women were found (Figure 2 and Supplementary Table 1). Looking at age-specific baseline prevalence, detection rates for MCPyV and HPyV10 were comparable in all age groups, while baseline prevalence rates for HPyV6, and less pronounced for HPyV7 and STLPyV, rose at both collection sites in the small group (*n* = 13) of participants aged ≥ 60 years (Supplementary Table 2A).

### Site-Specific Human Polyomavirus Period Prevalence and Persistence

**FIGURE 3:**
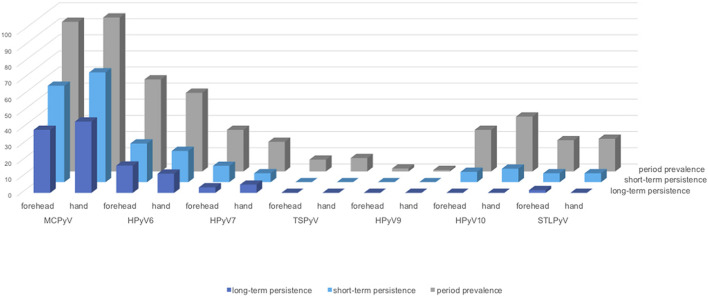
Human polyomavirus period-prevalence, short-, and long-term persistence by collection site. Y-axis: prevalence in percentage (%). Dark-blue bars: long-term persistence; light-blue bars: short-term persistence; grey bars: period prevalence (period-1).

Within collection period-1, MCPyV was the most prevalent HPyV (forehead 92.7%, 95%CI 86.0–96.8; hand 95.4%, 95%CI 89.6–98.5), followed by HPyV6 (forehead 56.9%, 95%CI 47.0–66.3; hand 48.6%, 95%CI 38.9–58.4) and, in decreasing order, by HPyV10, HPyV7, STLPyV, TSPyV, and HPyV9 (Table 3 and Figure 3). MCPyV, TSPyV, and HPyV10 period prevalence rates were comparable in all age groups at both sampling sites, while they tended to be higher in participants aged 60 years and older for HPyV6, HPyV7, and less prominent, in STLPyV (Supplementary Table 2B).

**TABLE 3:** Site-specific HPyV period-prevalence and short-term persistence.

Polyoma-virus	Forehead *n* = 109		Hand *n* = 109	
	Period prevalence No. (%, 95%CI^#^)	Short-term persistence No. (%, 95%CI^#^)	Period prevalence No. (%, 95%CI^#^)	Short-term persistence No. (%, 95%CI^#^)
MCPyV	101 (92.7, 86.0–96.8)	65 (59.6, 49.8–68.9)	104 (95.4, 89.6–98.5)	74 (67.9, 58.3–76.5)
HPyV6	62 (56.9, 47.0–66.3)	26 (23.9, 16.2–33.0)	53 (48.6, 38.9–58.4)	21 (19.3, 12.3–27.9)
HPyV7	28 (25.7, 17.8–34.9)	11 (10.1, 5.1–17.3)	20 (18.3, 11.6–26.9)	6 (5.5, 2.0–11.6)
TSPyV	8 (7.3, 3.2–14.0)	0 (0.0, 0.0–3.3)	9 (8.3, 3.8–15.1)	0 (0.0, 0.0–3.3)
HPyV9	2 (1.8, 0.2–6.5)	0 (0.0, 0.0–3.3)	1 (0.9, 0.0–5.0)	0 (0.0, 0.0–3.3)
HPyV10	28 (25.7, 17.8–34.9)	7 (6.4, 2.6–12.8)	37 (33.9, 25.1–43.6)	9 (8.3, 3.8–15.1)
STLPyV	21 (19.3, 12.3–27.9)	6 (5.5, 2.0–11.6)	22 (20.2, 13.1–28.0)	6 (5.5, 2.0–11.6)

*No., number; CI, confidence interval.*

*^#^Exact Clopper–Pearson confidence intervals.*

*Short-term persistence was defined as ≥3 of 4 swabs positive for the respective HPyV in collection period-1.*

Concerning short-term persistence, the highest rate was found for MCPyV with 59.6% (95%CI 49.8–68.9) on the forehead and 67.9% (95%CI 58.3–76.5) on the hand, followed by HPyV6 [forehead 23.9% (95%CI 16.2–33.0); hand 19.3% (95%CI 12.3–27.9)]. Short-term persistence rates at 10% or below were found for HPyV7, HPyV10, and STLPyV. No short-term persistence could be observed for TSPyV and HPyV9 (Table 3 and Figure 3).

Of the 59 volunteers available in collection period-2, around 60% had long-term persistence for any HPyV. Again, MCPyV was the most persistent virus with 39.0% (95%CI 26.5–52.6) on the forehead and 44.1% (95%CI 31.2–57.6) on the hand, followed by HPyV6 (forehead 16.9%, 95%CI 8.4–29.0; hand 11.9%, 95%CI 4.9–22.9) and HPyV7 (forehead 3.4%, 95%CI 0.4–11.7; hand 5.1%, 95%CI 1.1–14.1). Only one person had a long-term persistence for STLPyV on the forehead (1.7%; 95%CI 0.0–9.1). No long-term persistence occurred for TSPyV, HPyV9, and HPyV10 (Table 4 and Figure 3).

**TABLE 4:** Site-specific HPyV long-term persistence.

Polyomavirus	Forehead *n* = 59 No. (%, 95%CI^#^)	Hand *n* = 59 No. (%, 95%CI^#^)
MCPyV	23 (39.0, 26.5–52.6)	26 (44.1, 31.2–57.6)
HPyV6	10 (16.9, 8.4–29.0)	7 (11.9, 4.9–22.9)
HPyV7	2 (3.4, 0.4–11.7)	3 (5.1, 1.1–14.1)
TSPyV	0 (0.0, 0.0–6.1)	0 (0.0, 0.0–6.1)
HPyV9	0 (0.0, 0.0–6.1)	0 (0.0, 0.0–6.1)
HPyV10	0 (0.0, 0.0–6.1)	0 (0.0, 0.0–6.1)
STLPyV	1 (1.7, 0.0–9.1)	0 (0.0, 0.0–6.1)

*No., number; CI, confidence interval.*

*^#^Exact Clopper–Pearson confidence intervals.*

*Long-term persistence was defined as a positive follow-up swab in collection period-2 in volunteers with previous short-term persistence.*

### Human Polyomavirus DNA Persistence and Viral Load

**FIGURE 4:**
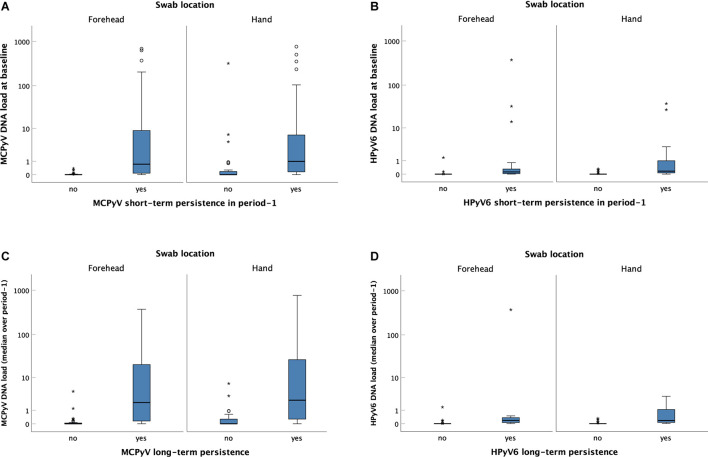
Merkel cell polyomavirus (MCPyV) and HPyV6 DNA loads on the forehead and the hand of individuals without and with short-term **(A,B)** and long-term **(C,D)** persistence. Viral DNA loads (Y-axis) were expressed as viral DNA copies per betaglobin-gene copy. Boxes represent the interquartile range with the median. Whiskers are vertical lines ending in horizontal lines at the largest and smallest observed values that are not statistical outliers, i.e., values more than three IQRs from the end of a box are labelled as extreme, denoted with an asterisk (*) and values more than 1.5 IQRs but less than 3 IQRs from the end of the box are labelled as outliers (o). **(A)** MCPyV and **(B)** HPyV6 baseline viral DNA load distribution of individuals without (left box plot) and with (right box plot) short-term persistence in collection period-1. The differences in viral DNA loads between individuals without and with short-term persistence were significant for both locations and both viruses [*p* < 0.001 for forehead (*n* = 109) and hand (*n* = 109), respectively; independent-samples Mann–Whitney *U* test]. **(C)** MCPyV and **(D)** HPyV6 median DNA loads over collection period-1 of individuals without (left box plot) and with (right box plot) long-term persistence. The differences in viral DNA loads between individuals without and with long-term persistence were significant for both locations and both viruses [*p* < 0.001 for forehead (*n* = 59) and hand (*n* = 59), respectively; independent-samples Mann–Whitney *U* test].

Volunteers with short-term persistence of MCPyV, HPyV6, HPyV7, HPyV10, and STLPyV had significantly higher viral DNA loads in the baseline swab compared to those without short-term persistence. This was true for both sampling sites (*p* < 0.001, respectively; Figures 4A,B and Supplementary Figures 1A–C). Similarly, participants with long-term persistence of MCPyV, HPyV6, and HPyV7 had significantly higher median viral loads in collection period-1 on forehead and hand, compared to those without long-term persistence (Figures 4C,D and Supplementary Figure 2).

### Risk Factors for Human Polyomavirus DNA Prevalence and Persistence

Evaluation of age, sex, smoking, underlying disease, and HPyV multiplicity at baseline as risk factors for prevalence and persistence of the different HPyVs was performed by univariable and multivariable logistic regression analyses. Regarding the forehead, significant risk factors for baseline prevalence were older age for HPyV6, male sex for MCPyV and multiplicity for all HPyVs with the exception of TSPyV and HPyV9 (Table 5). Multiplicity turned out to be a risk factor only for HPyV6, 7, and 10 period prevalence (Table 6). Risk factors for short-term persistence were older age for HPyV6, male sex for MCPyV and multiplicity for MCPyV, HPyV6, 7 and 10 (Table 7). Finally, risk factors for long-term persistence were only older age for HPyV6 and multiplicity for MCPyV and HPyV6 on the forehead (Table 8). Similar results were obtained for the hand (Supplementary Tables 3A–D). Smoking did not play any role as a risk factor. Underlying disease reduced the risk for MCPyV period prevalence on the forehead (OR 0.12, 95%CI 0.02–0.82, *p* = 0.030), but not on the hand (Table 6 and Supplementary Table 3B).

**TABLE 5:** Risk factors for HPyVs baseline prevalence on the forehead of healthy individuals found in univariable logistic regression analysis.

		MCPyV		HPyV6		HPyV7		TSPyV		HPyV9		HPyV10		STLPyV	
Candidate variables	*n*	OR (95% CI^#^)	*p*-value^1^	OR (95% CI^#^)	*p*-value^1^	OR (95% CI^#^)	*p*-value^1^	OR (95% CI^#^)	*p*-value^1^	OR (95% CI^#^)	*p*-value^1^	OR (95% CI^#^)	*p*-value^1^	OR (95% CI^#^)	*p*-value^1^
Age (per decade)	109	1.33 (0.96–1.85)	0.092	**1.91** (1.35– 2.70)	**<0.001**	1.34 (0.93– 1.93)	0.116	1.13 (0.30–4.31)	0.857	0.16 (0.01–4.95)	0.292	0.92 (0.59–1.43)	0.711	1.36 (0.86–2.17)	0.191
Sex (male vs. female)	109	**2.89** (1.19–7.00)	**0.019**	1.12 (0.49– 2.54)	0.785	1.23 (0.41– 3.69)	0.706	0 (0)	0.998	>1 (0)	0.997	0.57 (0.17–1.98)	0.378	0.18 (0.02–1.50)	0.112
Smoking (yes vs. no)	109	1.09 (0.42–2.81)	0.867	0.76 (0.29– 2.04)	0.591	0.45 (0.09–2.13)	0.314	>1 (0)	0.997	0 (0)	0.998	0.55 (0.11–2.64)	0.451	2.04 (0.45–9.17)	0.355
Underlying disease (yes vs. no)	109	0.45 (0.01–2.36)	0.345	1.11 (0.19– 6.37)	0.907	1.27 (0.14–11.70)	0.832	0 (0)	0.999	0 (0)	0.999	1.52 (0.16–14.10)	0.714	2.74 (0.28–26.82)	0.386
Multiplicity^2^ (yes vs. no)	109	**52.76** (6.84–406.81)	**<0.001**	**69.71** (14.91– 325.88)	**<0.001**	**27.13** (3.41–215.63)	**0.002**	>1 (0)	0.997	>1 (0)	0.997	**21.88** (2.73–175.58)	**0.004**	**11.13** (1.32–93.95)	**0.027**

*OR, odds ratio; CI, confidence interval.*

*^#^Exact Clopper–Pearson confidence intervals.*

*^1^*p*-values < 0.05 are significant and printed in bold.*

*^2^Defined as ≥two different HPyVs at baseline.*

*The risk factors found for MCPyV, HPyV6, HPyV7, HPyV10, and STLPyV in univariable regression analysis were included in a multivariable logistic regression model and remained significant after adjustment for the other covariates.*

**TABLE 6:** Risk factors for HPyVs period prevalence on the forehead of healthy individuals found in univariable logistic regression analysis.

		MCPyV		HPyV6		HPyV7		TSPyV		HPyV9		HPyV10		STLPyV	
Candidate variables	*n*	OR (95% CI^#^)	*p*-value^1^	OR (95% CI^#^)	*p*-value^1^	OR (95% CI^#^)	*p*-value^1^	OR (95% CI^#^)	*p*-value^1^	OR (95% CI^#^)	*p*-value^1^	OR (95% CI^#^)	*p*-value^1^	OR (95% CI^#^)	*p*-value^1^
Age (per decade)	109	0.87 (0.53–1.41)	0.563	**1.44** (1.05–1.97)	0.024*	1.30 (0.96– 1.76)	0.096	0.86 (0.48– 1.53)	0.603	0.71 (0.20–2.54)	0.600	0.88 (0.63– 1.23)	0.448	1.29 (0.93–1.79)	0.130
Sex (male vs. female)	109	5.63 (0.67–47.42)	0.112	0.72 (0.33– 1.55)	0.397	0.69 (0.29– 1.69)	0.421	1.41 (0.33– 5.94)	0.644	>1 (0)	0.997	**0.36** (0.14– 0.94)	**0.036**	0.48 (0.17–1.35)	0.165
Smoking (yes vs. no)	109	0.49 (0.11– 2.21)	0.355	2.53 (0.96–6.65)	0.061	0.62 (0.21–1.84)	0.391	2.04 (0.45– 9.17)	0.355	0 (0)	0.998	1.40 (0.53– 3.70)	0.498	2.39 (0.86–6.65)	0.094
Underlying disease (yes vs. no)	109	**0.12** (0.02–0.82)	**0.030**	0.36 (0.06–2.05)	0.248	0.56 (0.06– 5.04)	0.607	0 (0)	0.999	0 (0)	0.999	3.12 (0.59– 16.45)	0.180	2.21 (0.38–12.96)	0.379
Multiplicity^2^ (yes vs. no)	109	>1 (0)	0.997	**16.40** (5.65– 47.62)	**<0.001**	**4.22** (1.69– 10.57)	**0.002**	4.58 (0.88– 23.80)	0.071	>1 (0)	0.997	**2.77** (1.15– 6.71)	**0.024**	1.67 (0.64–4.34)	0.296

*OR, odds ratio; CI, confidence interval.*

*^#^Exact Clopper–Pearson confidence intervals.*

*^1^*p*-values < 0.05 are significant and printed in bold.*

*^2^Defined as ≥2 different HPyVs at baseline.*

*Not significant anymore in multivariable modeling.

*The risk factors found for MCPyV, HPyV6, HPyV7, HPyV10, and STLPyV in univariable regression analysis were included in a multivariable logistic regression model and remained significant after adjustment for the other covariates, with the exception of age for HPyV6.*

**TABLE 7:** Risk factors for HPyVs short-term persistence on the forehead of healthy individuals found in univariable logistic regression analysis.

		MCPyV		HPyV6		HPyV7		HPyV10		STLPyV	
Candidate variables	*n*	OR (95% CI^#^)	*p*-value^1^	OR (95% CI^#^)	*p*-value^1^	OR (95% CI^#^)	*p*-value^1^	OR (95% CI^#^)	*p*-value^1^	OR (95% CI^#^)	*p*-value^1^
Age (per decade)	109	1.28 (0.94–1.73)	0.114	**1.88** (1.33–2.67)	**<0.001**	**1.74** (1.15–2.62)	0.009*	0.99 (0.57–1.74)	0.981	1.64 (0.98–2.74)	0.059
Sex (male vs. female)	109	**2.93** (1.28–6.66)	**0.011**	0.82 (0.33–2.01)	0.658	1.16 (0.33–4.06)	0.818	1.03 (0.22–4.84)	0.971	0.26 (0.03–2.29)	0.223
Smoking (yes vs. no)	109	1.11 (0.45–2.74)	0.821	0.95 (0.33–2.68)	0.915	0.29 (0.04–2.40)	0.252	1.30 (0.24–7.13)	0.763	0 (0)	0.998
Underlying disease (yes vs. no)	109	0.32 (0.06–1.81)	0.197	0.62 (0.07–5.60)	0.674	1.86 (0.20–17.54)	0.588	3.23 (0.32–32.25)	0.317	3.92 (0.38–40.19)	0.250
Multiplicity^2^ (yes vs. no)	109	**5.14** (2.13–12.41)	**<0.001**	**33.27** (7.26–152.53)	**<0.001**	**7.42** (1.52–36.22)	**0.013**	**9.30** (1.08–80.16)	**0.042**	2.91 (0.51–16.59)	0.230

*OR, odds ratio; CI, confidence interval.*

*^#^Exact Clopper–Pearson confidence intervals.*

*^1^*p*-values < 0.05 are significant and printed in bold.*

*^2^Defined as ≥2 different HPyVs at baseline.*

*Not significant anymore in multivariable modeling.

*Short-term persistence did not occur in TSPyV and HPyV9. The risk factors found for MCPyV, HPyV6, HPyV7 and HPyV10 in univariable regression analysis were included in a multivariable logistic regression model and remained significant after adjustment for the other covariates, with the exception of age for HPyV7.*

**TABLE 8:** Risk factors for HPyVs long-term persistence on the forehead of healthy individuals found in univariable logistic regression analysis.

		MCPyV		HPyV6		HPyV7		STLPyV	
Candidate variables	*n*	OR (95% CI^#^)	*p*-value^1^	OR (95% CI^#^)	*p*-value^1^	OR (95% CI^#^)	*p*-value^1^	OR (95% CI^#^)	*p*-value^1^
Age (per decade)	109	1.34 (0.87–2.07)	0.186	**3.37** (1.64–6.92)	**0.001**	2.57 (0.91–7.22)	0.074	4.72 (0.55–40.81)	0.159
Sex (male vs. female)	109	2.48 (0.84– 7.32)	0.100	1.05 (0.26–4.22)	0.942	0 (0)	0.998	0 (0)	0.998
Smoking (yes vs. no)	109	0.53 (0.12–2.23)	0.382	0.43 (0.05–3.83)	0.452	0 (0)	0.999	0 (0)	0.999
Underlying disease (yes vs. no)	109	0 (0)	1.000	>1 (0)	1.000	0 (0)	1.000	0 (0)	1.000
Multiplicity^2^ (yes vs. no)	109	**3.75** (1.25–11.30)	**0.019**	**15.50** (1.81–132.54)	**0.012**	>1 (0)	0.998	>1 (0)	0.998

*OR, odds ratio; CI, confidence interval.*

*^#^Exact Clopper–Pearson confidence intervals.*

*^1^*p*-values < 0.05 are significant.*

*^2^Defined as ≥2 different HPyVs at baseline.*

*Long-term persistence did not occur in TSPyV, HPyV9, and HPyV10. The risk factors found for MCPyV and HPyV6 in univariable regression analysis were included in a multivariable logistic regression model and remained significant after adjustment for the other covariates.*

## Discussion

In the present study, we have analysed prevalence, short- and long-term persistence of human polyomaviruses MCPyV, HPyV6, HPyV7, TSPyV, HPyV9, HPyV10, and STLPyV on healthy skin. HPyV12, NJPyV-2013, LIPyV, and QPyV were not included in the present study due to their low seroprevalence, the uncertainty that they are genuine HPyVs and/or their unknown pathogenicity in humans (Nicol et al., 2013; Van Der Meijden et al., 2013; Gossai et al., 2016; Moens et al., 2017; Kamminga et al., 2018).

The HPyV DNA detection rates at baseline observed here are basically in line with previous studies. Hampras et al. (2015) detected MCPyV, HPyV6, and HPyV7 in 65.9, 12, and 2.1% of 192 healthy males, respectively. In previous studies from our group MCPyV, HPyV6, HPyV7, TSPyV, HPyV9, and HPyV10 were found in 49.4, 27.6, 13.4, 0.8, 0, and 3.4% of healthy males who underwent skin cancer screening, respectively (Wieland et al., 2011, 2014). A study from Japan reported comparable prevalence rates for MCPyV, HPyV6, and HPyV7 in healthy individuals (Hashida et al., 2016, 2018). The low baseline prevalence for TSPyV and HPyV9 found in the present study is consistent with the results from recent studies, in which both viruses were either undetectable or were detected only sporadically on the skin of adults (Wieland et al., 2014; Hampras et al., 2015; Wang et al., 2019). Interestingly, here, we observed a higher HPyV10 DNA prevalence (11.9%) compared to 3.4% of healthy males in a former study (Wieland et al., 2014). The difference might be related to the inclusion of women, as in the present study, HPyV10 baseline prevalence was higher in women compared to men, however, without reaching significance. To the best of our knowledge, this is the first study to provide data on prevalence of STLPyV in the skin, which was found in 7.3 and 8.3% on forehead and hand at baseline, respectively. STLPyV was first detected in stool specimens (Lim et al., 2013). Recently, STLPyV was found in 2% of palatine tonsil swabs from children with chronic tonsillar disease (Peng et al., 2016), while in another study, it was undetectable in benign and malignant tonsillar tissue of adults (Herberhold et al., 2017). STLPyV was not detected in malignant and non-malignant lung tissue (Csoma et al., 2018) but was found in nose and throat swabs from a kidney transplant recipient (Bialasiewicz et al., 2016) and in skin warts from the buttocks of a patient suffering from a primary immunodeficiency (Pastrana et al., 2013).

Baseline viral loads measured here are comparable to data previously published (Wieland et al., 2011, 2014; Hashida et al., 2016, 2018). With the exception of MCPyV, HPyV DNA loads in non-lesional skin swabs were low, possibly indicating chronic shedding of HPyV from healthy skin with low-level replication, similar to cutaneous human papillomaviruses of the genus beta (Weissenborn et al., 2009, 2010; Schowalter et al., 2010).

In contrast to Foulongne et al. (2010b) who found a significantly higher MCPyV DNA prevalence on the face compared to limbs and Hampras et al. (2015) who found higher MCPyV detection rates in arm swabs compared to eyebrow hair, we could not observe site-specific differences regarding baseline prevalence rates of all HPyVs analysed. This is in line with a study showing that the amount of MCPyV DNA shed from the skin is similar at multiple environmentally exposed and unexposed anatomical sites (Pastrana et al., 2012). However, we observed higher MCPyV, HPyV6, HPyV10, and STLPyV DNA loads at baseline in hand compared to forehead swabs. Possibly, an increased rate of cell division in environmentally exposed areas of the body might favour amplification of commensal viruses.

Investigating consecutive skin swabs showed that above 90% of participants were MCPyV-positive, about half HPyV6-positive and about a quarter HPyV7-positive within the 12–18 weeks of period-1. The higher prevalence rates in period-1 compared to baseline are in line with incident infections of MCPyV, HPyV6, and HPyV7 in follow-up samples as reported by Hampras et al. (2015).

Short-term persistence was observed on both locations in over two-thirds of MCPyV-positive cases, which is in line with persistence rates on healthy skin found by Hashida et al. (2016) in 38 individuals who were reassessed 6 months after baseline sample. Hampras et al. (2015) observed comparable MCPyV persistence rates in normal skin and eyebrow hairs of healthy males, while HPyV6 and HPyV7 persistence were detected more frequently (73.0 and 37.5%, respectively) than in the present work. However, the definition of short-term persistence used here (three of four swabs positive) was stricter than the definition used by Hampras et al. (2015) (two positive consecutive swabs), and both studies differ in terms of population and sampling intervals. So far, no comparable data has been published on persistence of the remaining HPyVs investigated here. While no short-term persistence was observed for TSPyV and HPyV9, polyomaviruses HPyV10 and STLPyV persisted in both locations in 6–8%, which has not yet been shown for STLPyV.

Long-term persistence was observed for MCPyV in 39.0% (forehead) and 44.1% (hand), followed by HPyV6 (16.9 and 11.9%), HPyV7 (3.4 and 5.1%) and, in one case, STLPyV, while it did not occur in the other HPyVs investigated. However, a single negative follow-up swab does not definitely rule out long-term persistence, especially when short-term persistent individuals were lost to follow-up. A reduction of HPyV-positive individuals in collection period-2 (2021) might also be explained by frequent disinfection of the hands and contact reduction due to the COVID-19 pandemic. The finding that persistent infections with TSPyV did not occur supports the assumption that the clinical picture of trichodysplasia spinulosa is the result of primary TSPyV infection (Van Der Meijden et al., 2017; Borgogna et al., 2019).

Elevated HPyV DNA loads were associated both with short- and long-term persistence of the HPyVs investigated in the present study. This is in line with results from Hashida et al. (2016) for MCPyV’s 6-month persistence and might be of clinical relevance. Possibly elevated cutaneous MCPyV loads could indicate an increased risk for later MCC development, since higher MCPyV loads are associated with long-term persistence and thus with possibly sustained oncogene expression and more chances for the viral genome to integrate into the host genome.

The major risk factor for baseline prevalence and short-term persistence of MCPyV, HPyV6, HPyV7, and HPyV10, as well as for long-term persistence of MCPyV and HPyV6 DNA found in multivariable regression analysis, was the detection of two or more HPyVs per swab (multiplicity) at baseline. Waning immunity in aging people may lead to enhanced viral replication. Concordantly, baseline prevalence for HPyV6, HPyV7, and STLPyV rose in participants aged 60 years and older. However, in multivariable regression analyses, older age remained only a risk factor for baseline prevalence, short- and long-term persistence of HPyV6. Increasing MCPyV, HPyV6, and HPyV7 prevalence with age has been described by other groups in eyebrow hairs but not in normal skin (Hampras et al., 2015; Hashida et al., 2016, 2018).

Male sex was an independent risk factor for MCPyV baseline prevalence and short-term persistence. According to literature, men are more likely to develop MCC than women (Stang et al., 2018). However, considering the widespread distribution of MCPyV, MCC development seems to be a rare multifactorial event with several risk factors such as UV radiation, older age and immunosuppression and is probably not solely due to the persistence of MCPyV in the skin (Foulongne et al., 2010a). Smoking was shown to be a risk factor for persistent human papillomavirus infections and subsequent development of anogenital warts, precancer, and cancer (Arcavi and Benowitz, 2004; Kaderli et al., 2014; Wieland et al., 2015). In contrast, current smoking was not associated with MCPyV prevalence in previous studies (Wieland et al., 2011; Hampras et al., 2015). This is in line with the present study showing that smoking was not a risk factor for prevalence or persistence of any HPyV.

Our results on prevalence and persistence of MCPyV, HPyV6, and HPyV7 are in concordance with serological studies in healthy individuals that found antibodies against MCPyV, HPyV6, and HPyV7 capsid proteins in 81.9, 83.8, and 71.7% of healthy blood donors, respectively (Kamminga et al., 2018). TSPyV- and HPyV9-specific antibodies were detected in 80–90% and >30–70%, respectively, and primary infection seems to occur during early childhood and adolescence (Foulongne et al., 2012; Nicol et al., 2013; Kamminga et al., 2018). Hence, it is possible that the skin is not the only tissue infected by these viruses. This notion is supported by several studies, in which TSPyV and HPyV9 were detected sporadically in tonsil tissue, faeces, respiratory samples, urine or peripheral blood, mainly in immunosuppressed persons (Rockett et al., 2013; Herberhold et al., 2017; Kourieh et al., 2018; Borgogna et al., 2019; Kamminga et al., 2019). In our study, short-term persistence rates of HPyV10 were low and long-term persistence did not occur, although HPyV10 reaches a seroprevalence rate of nearly 100% in adults (Kamminga et al., 2018). This indicates that the skin is only transiently infected and does not represent a permanent viral reservoir of HPyV10. HPyV10/MWPyV has been found in respiratory specimens, in 18.4% of tonsil cancer samples and in 5–7% of stool specimens from children and adults (Rockett et al., 2013; Herberhold et al., 2017; Pinheiro et al., 2020). The relatively high overall seroprevalence of STLPyV (64.8%) compared to the low DNA prevalence and persistence rate detected in our study also suggests sites of infection other than the skin, particularly the gut (Lim et al., 2013; Kamminga et al., 2018).

Limitations of our study are the high number of participants lost to long-term follow-up, and the fact that only a single swab pair was sampled in collection period-2. Furthermore, the number of participants 60 years and older was smaller than the number of participants in the other age groups and the sample was not representative of the German general population. We have not performed sequence analysis of the viral isolates and thus cannot exclude that persistent detection of the same HPyV represents new infections with different isolates instead of persistence of the same isolate. To corroborate our findings, future studies should include a larger number of participants of all ages and sexes, more frequent swab sampling during long-term follow-up and next-generation sequencing of individual samples. A strength of this study is that in contrast to previous studies (Wieland et al., 2011, 2014; Hampras et al., 2015), not only swabs from men but also from healthy women were included. Furthermore, the present study allowed comparison of HPyV prevalence, persistence and viral load in paired skin samples from two different body sites. To the best of our knowledge, this is the first study to collect long-term data over a decade on cutaneous HPyVs.

In summary, long-term persistence on the skin was shown for MCPyV, HPyV6, and HPyV7 on both forehead and hand. Higher viral loads and HPyV multiplicity were associated with short- and long-term persistence for several of the analysed HPyVs. In contrast to MCPyV, HPyV6, and HPyV7 and to a lesser extent STLPyV, polyomaviruses TSPyV and HPyV9 do not seem to be a regular part of the human skin virome in healthy individuals.

## Data Availability Statement

The raw data supporting the conclusion of this article will be made available by the authors, without undue reservation.

## Ethics Statement

The studies involving human participants were reviewed and approved by Ethics review board of the faculty of medicine of the University of Cologne. The participants provided their written informed consent to participate in this study.

## Author Contributions

LB wrote the manuscript and made contributions to the analysis and interpretation of data. MH performed the statistical analyses. AK and HP made substantial contributions to sampling and critically revising the manuscript for important intellectual content. UW and SS designed the study, performed sampling, analysis and interpretation of data, and drafted the manuscript. All authors contributed to the article and approved the submitted version.

## Conflict of Interest

The authors declare that the research was conducted in the absence of any commercial or financial relationships that could be construed as a potential conflict of interest.

## Publisher’s Note

All claims expressed in this article are solely those of the authors and do not necessarily represent those of their affiliated organizations, or those of the publisher, the editors and the reviewers. Any product that may be evaluated in this article, or claim that may be made by its manufacturer, is not guaranteed or endorsed by the publisher.
