# Supplementary material for: Natural History of Cutaneous Human Polyomavirus Infection in Healthy Individuals
Source: Front Microbiol. 2021 Oct 18;12:740947. doi: 10.3389/fmicb.2021.740947 (PMC8558461; doi:10.3389/fmicb.2021.740947)
Supplement: Supplementary file 1 [file Data_Sheet_1.docx]

**
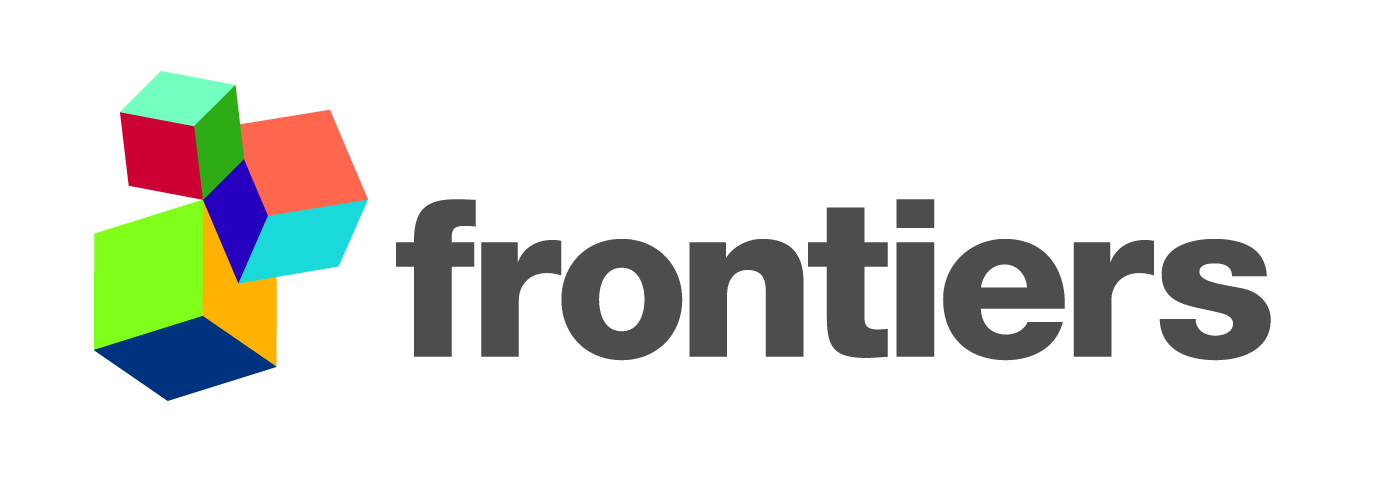
*Supplementary Material***

1. **Supplementary Tables**

**Table S1 Site-specific HPyV baseline prevalence by sex**

| **Sex** | n = 109 | **MCPyV**  No. (%, 95%CI^#^) | | **HPyV6**  No. (%, 95%CI^#^) | | **HPyV7**  No. (%, 95%CI^#^) | | **TSPyV**  No. (%, 95%CI^#^) | | **HPyV9**  No. (%, 95%CI^#^) | | **HPyV10**  No. (%, 95%CI^#^) | | **STLPyV**  No. (%, 95%CI^#^) | |
| --- | --- | --- | --- | --- | --- | --- | --- | --- | --- | --- | --- | --- | --- | --- | --- |
|  | No. (%) | fore-head | hand | fore-head | hand | fore-head | hand | fore-head | hand | fore-head | hand | fore-head | hand | fore-head | hand |
| female | 63 (57.8) | 37 (58.7, CI 45.6-71.0) | 36 (57.1, CI 44.0-69.5) | 19 (30.2, CI 19.2-43.0) | 17 27.0, CI 16.6-39.7) | 8 (12.7, CI 5.6-23.5) | 10 (15.9, CI 7.9-27.3) | 1 (1.6, CI 0.0-8.5) | 1 (1.6, CI 0.0-8.5) | 0 (0, CI 0.0-5.7) | 0 (0, CI 0.0-5.7) | 9 (14.3, CI 6.7-25.4) | 11 (17.5, CI 9.1-29.1) | 7  (11.1, CI 4.6-21.6) | 7 (11.1, CI 4.6-21.6) |
| male | 46 (42.2) | 37 (80.4, CI 66.1-90.6) | 37 (80.4, CI 66.1-90.6) | 15 (32.6, CI 19.5-48.0) | 11 (23.9  , CI 12.6-38.8) | 7 (15.2, CI 6.3-28.9) | 2 (4.3, CI 0.5-14.8) | 0 (0 CI 0.0-7.7) | 0 (0, CI 0.0-7.7) | 1 (2.2, CI 0.1-11.5) | 1 (2.2, CI 0.1-11.5) | 4 (8.7, CI 2.4-20.8) | 6 (13.0, CI 4.9-26.3) | 1 (2.2, CI 0.1-11.5) | 2 (4.3, CI 0.5-14.8) |

No., number; CI, confidence interval; ^#^Exact Clopper-Pearson confidence intervals

**Table S2A Age-specific prevalence at baseline by collection site**

| **Age** (yr) |  | **MCPyV**  No. (%, 95%CI^#^) | | **HPyV6**  No. (%, 95%CI^#^) | | **HPyV7**  No. (%, 95%CI^#^) | | **TSPyV**  No. (%, 95%CI^#^) | | **HPyV9**  No. (%, 95%CI^#^) | | **HPyV10**  No. (%, 95%CI^#^) | | **STLPyV**  No. (%, 95%CI^#^) | |
| --- | --- | --- | --- | --- | --- | --- | --- | --- | --- | --- | --- | --- | --- | --- | --- |
|  | No. (%) | fore-head | hand | fore-head | hand | fore-head | hand | fore-head | hand | fore-head | hand | fore-head | hand | fore-head | hand |
| 20 -39 | 55 (50.4) | 35 (63.6, 49.6-76.2) | 34 (61.8, 47.7-74.6) | 10 (18.2, 9.1-30.9) | 9 (16.4, 7.8-28.8) | 4 (7.3, 2.0-17.6) | 6 (10.9, 4.1-22.2) | 0 (0, 0.0-6.5) | 1 (1.8, 0.0-9.7) | 1 (1.8, 0.0-9.7) | 1 (1.8, 0.0-9.7) | 8 (14.5, 6.5-26.7) | 10 (18.2, 9.1-30.9) | 4 (7.3, 2.0-17.6) | 4 (7.3, 2.0-17.6)) |
| 40 - 59 | 41 (37.6) | 28 (68.3, 51.9-81.9) | 30 (73.2, 57.1-85.8) | 14 (34.1, 20.1-50.6) | 11 (26.8, 14.2-42.9) | 7 (17.1, 7.2-32.1) | 2 (4.9, 0.6-16.5) | 1 (2.4, 0.1-12.9) | 0 (0, 0.0-8.6) | 0 (0, 0.0-8.6) | 0 (0, 0.0-8.6) | 3 (7.3, 1.5-19.9) | 4 (9.8, 2.7-23.1) | 2 (4.9, 0.6-16.5) | 1 (2.4, 0.1-12.9) |
| ≥ 60 | 13 (12) | 11 (84.6, 54.6-89.1 | 9 (69.2, 38.6-90.9) | 10 (76.9, 46.2-95.0) | 8 (61.5, 31.6-86.1) | 4 (30.8, 9.1-61.4) | 4 (30.8, 9.1-61.4) | 0 (0, 0.0-24.7) | 0 (0, 0.0-24.7) | 0 (0, 0.0-24.7) | 0 (0, 0.0-24.7) | 2 (15.4, 1.9-45.4) | 3 (23.1, 5.0-53.8) | 2 (15.4, 1.9-45.4) | 4 (30.8, 9.1-61.4) |
| Total | 109 (100) | 74 (67.9, 58.3-76.5) | 73 (67.0, 57.3-75.7) | 34 (31.2, 22.7-40.8) | 28 (25.7, 17.8-34.9) | 15 (13.8, 7.9-21.7) | 12 (11.0, 5.8-18.4) | 1 (0.9, 0-5.0) | 1 (0.9, 0-5.0) | 1 (0.9, 0-5.0) | 1 (0.9, 0-5.0) | 13 (11.9, 6.5-19.5) | 17 (15.6, 9.4-23.8) | 8 (7.3, 3.2-14.0) | 9 (8.3, 3.8-15.1) |

No., number; CI, confidence interval; ^#^Exact Clopper-Pearson confidence intervals; yr, year

**Table S2B Age-specific period prevalence (period 1) by collection site**

| **Age** (yr) |  | **MCPyV**  No. (%, 95%CI^#^) | | **HPyV6**  No. (%, 95%CI^#^) | | **HPyV7**  No. (%, 95%CI^#^) | | **TSPyV**  No. (%, 95%CI^#^) | | **HPyV9**  No. (%, 95%CI^#^) | | **HPyV10**  No. (%, 95%CI^#^) | | **STLPyV**  No. (%, 95%CI^#^) | |
| --- | --- | --- | --- | --- | --- | --- | --- | --- | --- | --- | --- | --- | --- | --- | --- |
|  | No. (% of total) | fore-head | hand | fore-head | hand | fore-head | hand | fore-head | hand | fore-head | hand | fore-head | hand | fore-head | hand |
| 20 -39 | 55 (50.4) | 51 (92.7, 82.4-98.0) | 51 (92.7, 82.4-98.0) | 27 (49.1, 35.4-62.9) | 24 (43.6, 30.3-57.7) | 11 (20.0, 10.4-33.0) | 8 (14.5, 6.5-26.7) | 5 (9.1, 3.0-20.0) | 5 (9.1, 3.0-20.0) | 1 (1.8, 0.0-9.7) | 1 (1.8, 0.0-9.7) | 16 (29.1, 17.6-42.9) | 23 (41.8, 28.7-55.9) | 9 (16.4, 7.8-28.8) | 10 (18.2, 9.1-30.9) |
| 40 - 59 | 41 (37.6) | 39 (95.1, 83.5-99.4) | 40 (97.6, 87.1-99.9) | 23 (56.1, 39.7-71.5) | 20 (48.8, 32.9-64.9) | 11 (26.8, 14.2-42.9) | 6 (14.6, 5.6-29.2) | 2 (4.9, 0.6-16.5) | 3 (7.3, 1.5-19.9) | 1 (2.4, 0.1-12.9) | 0 (0, 0.0-8.6) | 8 (19.5, 8.8-34.9) | 8 (19.5, 8.8-34.9) | 8 (19.5, 8.8-34.9) | 8 (19.5, 8.8-34.9) |
| ≥ 60 | 13 (12) | 11 (84.6, 54.6-98.1) | 13 (100, 75.3-100) | 12  (92.3, 64.0-99.8) | 9 (69.2, 38.6-90.9) | 6 (46.2, 19.2-74.9) | 6 (46.2, 19.2-74.9) | 1 (7.7, 0.2-36.0) | 1 (7.7, 0.2-36.0) | 0 (0, 0.0-24.7) | 0 (0, 0.0-24.7) | 4 (30.8, 9.1-61.4) | 6 (46.2, 19.2-74.9) | 4 (30.8, 9.1-61.4) | 4 (30.8, 9.1-61.4) |
| Total | 109 (100) | 101 (92.7, 86.0-96.8) | 104 (95.4, 89.6-98.5) | 62 (56.9, 47.0-66.3) | 53 (48.6, 38.9-58.4) | 28 (25.7, 17.8-34.9) | 20 (18.3, 11.6-26.9) | 8 (7.3, 3.2-14.0) | 9 (8.3, 3.8-15.1) | 2 (1.8, 0.2-6.5) | 1 (0.9, 0.0-5.0) | 28 (25.7, 17.8-34.9) | 37 (33.9, 25.1-43.6) | 21 (19.3, 12.3-27.9) | 22 (20.2, 13.1-28.0) |

No., number; CI, confidence interval; ^#^Exact Clopper-Pearson confidence intervals; yr, year

**Table S3A Risk factors for HPyVs baseline prevalence on the hand of healthy individuals found in univariable logistic regression analysis**

|  |  | **MCPyV** | | **HPyV6** | | **HPyV7** | | **TSPyV** | | **HPyV9** | | **HPyV10** | | **STLPyV** | |
| --- | --- | --- | --- | --- | --- | --- | --- | --- | --- | --- | --- | --- | --- | --- | --- |
| Candidate variables | n | OR  (95% CI^#^) | p-value^1^ | OR  (95% CI^#^) | p-value^1^ | OR  (95% CI^#^) | p-value^1^ | OR  (95% CI^#^) | p-value^1^ | OR  (95% CI^#^) | p-value^1^ | OR  (95% CI^#^) | p-value^1^ | OR  (95% CI^#^) | p-value^1^ |
| Age  (per decade) | 109 | 1.23  (0.90 - 1.68) | 0.200 | **1.91**  (1.35 - 2.70) | **<0.001*** | 1.24 (0.83 - 1.85) | 0.303 | 0.74  (0.13 -4.26) | 0.739 | 0.16  (0.01 - 4.95) | 0.292 | 0.91  (0.61 -  1.35) | 0.638 | **1.71**  (1.10 -  2.65) | **0.017*** |
| Sex  (male vs. female) | 109 | **3.08**  (1.28 - 7.46) | **0.012** | 0.85  (0.35 - 2.04) | 0.717 | 0.24 (0.05 – 1.16) | 0.076 | 0  (0) | 0.998 | > 1  (0) | 0.997 | 0.71  (0.24 -  2.08) | 0.531 | 0.36  (0.07 -  1.84) | 0.221 |
| Smoking  (yes vs. no) | 109 | 0.59  (0.24 -1.46) | 0.252 | 0.83 (0.30 - 2.34) | 0.727 | 0.61 (0.12 - 2.97) | 0.539 | > 1  (0) | 0.997 | 0  (0) | 0.998 | 0.98  (0.29 -  3.31) | 0.973 | 0.91  (0.18 -  4.65) | 0.905 |
| Underlying disease  (yes vs. no) | 109 | 0.99  (0.17 -5.65) | 0.987 | 0.56 (0.06 - 5.04) | 0.607 | 0  (0) | 0.999 | 0  (0) | 0.999 | 0  (0) | 0.999 | 2.93  (0.49 -  17.46) | 0.237 | **6.85**  (1.06 -  44.17) | **0.043** |
| Multiplicity^2^  (yes vs. no) | 109 | 2.43  (0.94 -6.30) | 0.067 | **66.67** (16.71 - 265.95) | **<0.001** | > 1  (0) | 0.996 | > 1  (0) | 0.997 | > 1  (0) | 0.997 | **16.80**  (4.39 -  64.27) | **<0.001** | **9.46**  (1.85 -  48.41) | **0.007** |

OR, Odds Ratio; CI, confidence interval; ^#^Exact Clopper-Pearson confidence intervals; ^1^p-values <0.05 are significant and printed in bold, ^2^defined as ≥ 2 HPyV types at baseline. * not significant anymore in multivariable modeling

The risk factors found in univariable regression analysis were included in a multivariable logistic regression model and remained significant, with the exception of age for HPyV6 and STLPyV.

**Table S3B Risk factors for HPyVs period prevalence on the hand of healthy individuals found in univariable logistic regression analysis**

|  |  | **MCPyV** | | **HPyV6** | | **HPyV7** | | **TSPyV** | | **HPyV9** | | **HPyV10** | | **STLPyV** | |
| --- | --- | --- | --- | --- | --- | --- | --- | --- | --- | --- | --- | --- | --- | --- | --- |
| Candidate variables | n | OR  (95% CI^#^) | p-value^1^ | OR  (95% CI^#^) | p-value^1^ | OR  (95% CI^#^) | p-value^1^ | OR  (95% CI^#^) | p-value^1^ | OR  (95% CI^#^) | p-value^1^ | OR  (95% CI^#^) | p-value^1^ | OR  (95% CI^#^) | p-value^1^ |
| Age  (per decade) | 109 | 1.60  (0.67 - 3.86) | 0.293 | 1.22 (0.92 - 1.63) | 0.162 | **1.42** (1.01 - 1.98) | **0.041** | 0.86 (0.50 - 1.49) | 0.594 | 0.16  (0.01 – 4.95) | 0.292 | 0.82 (0.60 - 1.12) | 0.216 | 1.27 (0.92 - 1.76) | 0.144 |
| Sex  (male vs. female) | 109 | 1.10  (0.18 - 6.86) | 0.919 | 0.95 (0.44 - 2.03) | 0.887 | 0.69 (0.25 - 1.90) | 0.472 | 0.66 (0.16 - 2.80) | 0.576 | > 1  (0) | 0.997 | **0.30** (0.13 - 0.73) | **0.008** | **0.33** (0.11 - 0.97) | **0.045** |
| Smoking  (yes vs. no) | 109 | 1.27  (0.14 - 11.86) | 0.836 | 0.72 (0.29 - 1.74) | 0.461 | 0.76 (0.23 - 2.52) | 0.655 | 1.67 (0.39 - 7.22) | 0.490 | 0  (0) | 0.998 | 0.83 (0.32 - 2.13) | 0.695 | 0.92 (0.30- 2.81) | 0.890 |
| Underlying disease  (yes vs. no) | 109 | 0.20  (0.02 - 2.16) | 0.186 | 2.20 (0.39 - 12.57) | 0.374 | 2.36 (0.40 - 13.89) | 0.342 | 0  (0) | 0.999 | 0  (0) | 0.999 | 0.97 (0.17 - 5.57) | 0.974 | 2.08 (0.36 - 12.14) | 0.418 |
| Multiplicity^2^  (yes vs. no) | 109 | > 1  (0) | 0.998 | **9.33** (3.42 - 25.47) | **<0.001** | **8.05** (2.74 - 23.66) | **<0.001** | **5.14** (1.20 - 21.99) | **0.027** | > 1  (0) | 0.997 | **3.32** (1.42 - 7.77) | **0.006** | **2.78** (1.06 - 7.28) | **0.037*** |

OR, Odds Ratio; CI, confidence interval; ^#^Exact Clopper-Pearson confidence intervals; ^1^p-values <0.05 are significant and printed in bold, ^2^defined as ≥ 2 HPyV types at baseline. * not significant anymore in multivariable modeling

The risk factors found in univariable regression analysis were included in a multivariable logistic regression model and remained significant after adjustment for the other covariates, with the exception of multiplicity for STLPyV.

**Table S3C Risk factors for HPyVs short-term persistence on the hand of healthy individuals found in univariable logistic regression analysis**

|  |  | **MCPyV** | | **HPyV6** | | **HPyV7** | | **HPyV10** | | **STLPyV** | |
| --- | --- | --- | --- | --- | --- | --- | --- | --- | --- | --- | --- |
| Candidate variables | n | OR  (95% CI^#^) | p-value^1^ | OR  (95% CI^#^) | p-value^1^ | OR  (95% CI^#^) | p-value^1^ | OR  (95% CI^#^) | p-value^1^ | OR  (95% CI^#^) | p-value^1^ |
| Age  (per decade) | 109 | 1.12 (0.83 - 1.52) | 0.461 | **1.52** (1.09 - 2.12) | **0.015*** | 1.54 (0.92 - 2.58) | 0.100 | 0.77 (0.43 - 1.38) | 0.377 | 1.64 (0.98 - 2.74) | 0.059 |
| Sex  (male vs. female) | 109 | **2.37** (1.00 - 5.62) | **0.050*** | 1.03 (0.40 - 2.71) | 0.946 | 0.67 (0.12 - 3.83) | 0.653 | 0.36 (0.07 - 1.84) | 0.221 | 0.26 (0.03 - 2.29) | 0.223 |
| Smoking  (yes vs. no) | 109 | 0.69 (0.28 - 1.73) | 0.428 | 0.71 (0.21 - 2.32) | 0.567 | 0.62 (0.07 - 5.60) | 0.674 | 1.67 (0.39 - 7.22) | 0.490 | 0  (0) | 0.998 |
| Underlying disease  (yes vs. no) | 109 | 0.94 (0.16 - 5.41) | 0.947 | 0.83 (0.09 – 7.51) | 0.868 | 3.92 (0.38 - 40.19) | 0.250 | 0  (0) | 0.999 | 3.92 (0.38 - 40.19) | 0.250 |
| Multiplicity^2^  (yes vs. no) | 109 | 1.47 (0.60 - 3.62) | 0.397 | **27.00** (7.09 - 102.80) | **<0.001** | > 1  (0) | 0.997 | **9.46** (1.85 - 48.41) | **0.007** | 4.87 (0.85 - 28.00) | 0.076 |

OR, Odds Ratio; CI, confidence interval; ^#^Exact Clopper-Pearson confidence intervals; ^1^p-values <0.05 are significant and printed in bold, ^2^defined as ≥ 2 HPyV types at baseline. * not significant anymore in multivariable modeling.

Short-term persistence did not occur in TSPyV and HPyV9. The risk factors found in univariable regression analysis were included in a multivariable logistic regression model and remained significant after adjustment for the other covariates, with the exception of male sex for MCPyV and age for HPyV6.

**Table S3D Risk factors for HPyVs long-term persistence on the hand of healthy individuals found in univariable logistic regression analysis**

|  |  | **MCPyV** | | **HPyV6** | | **HPyV7** | |
| --- | --- | --- | --- | --- | --- | --- | --- |
| Candidate variables | n | OR  (95% CI^#^) | p-value^1^ | OR  (95% CI^#^) | p-value^1^ | OR  (95% CI^#^) | p-value^1^ |
| Age  (per decade) | 109 | 1.06 (0.70 - 1.61) | 0.776 | **2.23**  (1.19 -  4.15) | **0.012*** | 1.75  (0.79 -  3.91) | 0.171 |
| Sex  (male vs. female) | 109 | **3.11** (1.05 - 9.23) | **0.041*** | 1.20  (0.24 -  5.93) | 0.823 | 0  (0) | 0.998 |
| Smoking  (yes vs. no) | 109 | 0.22 (0.04 -1.14) | 0.071 | 0.70  (0.08 -  6.49) | 0.754 | 2.30  (0.19 - 27.91) | 0.513 |
| Underlying disease  (yes vs. no) | 109 | 0  (0) | 1.000 | > 1  (0) | 1.000 | 0  (0) | 1.000 |
| Multiplicity^2^  (yes vs. no) | 109 | 0.60 (0.19 – 1.92) | 0.390 | **22.36**  (2.43 - 205.73) | **0.006** | > 1  (0) | 0.997 |

OR, Odds Ratio; CI, confidence interval; ^#^Exact Clopper-Pearson confidence intervals; ^1^p-values <0.05 are significant and printed in bold, ^2^defined as ≥ 2 HPyV types at baseline. * not significant anymore in multivariable modeling.

Long-term persistence did not occur in TSPyV, HPyV9, HPyV10 and STLPyV. The risk factors found in univariable regression analysis were included in a multivariable logistic regression model and only multiplicity for HPyV6 remained significant after adjustment for the other covariates.

1. **Supplementary Figures**

| 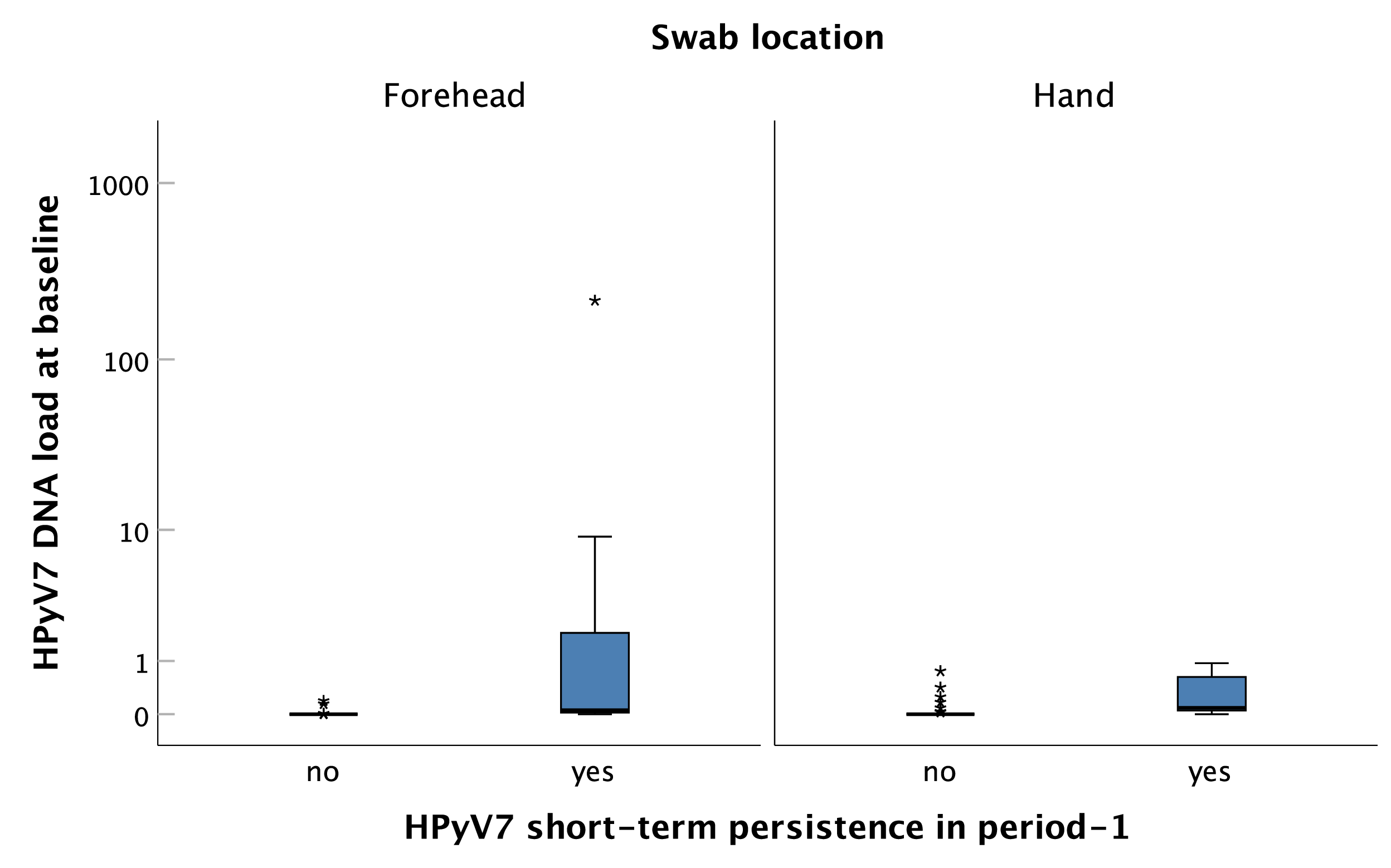  **A** |
| --- |
| 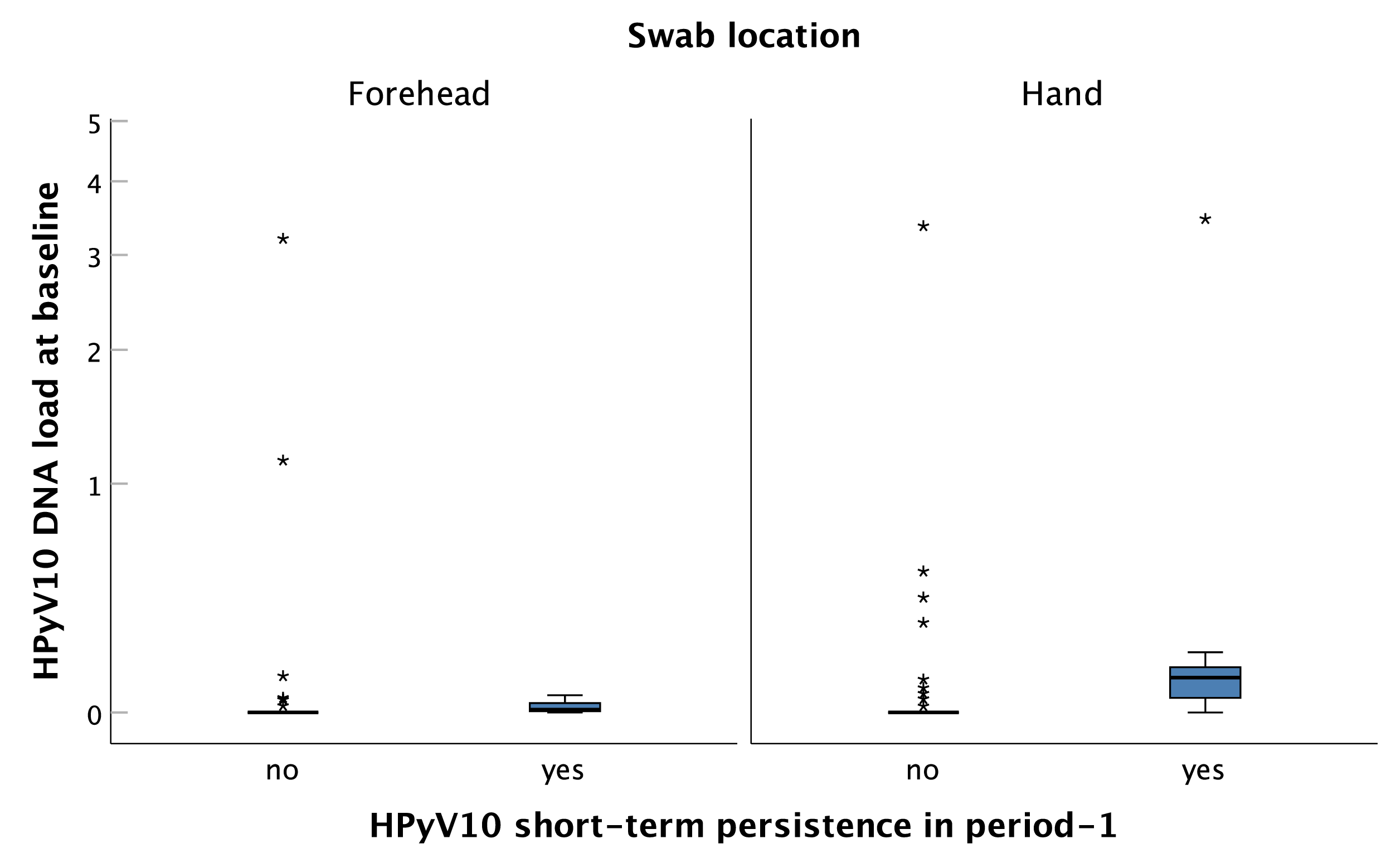  **B** |
| 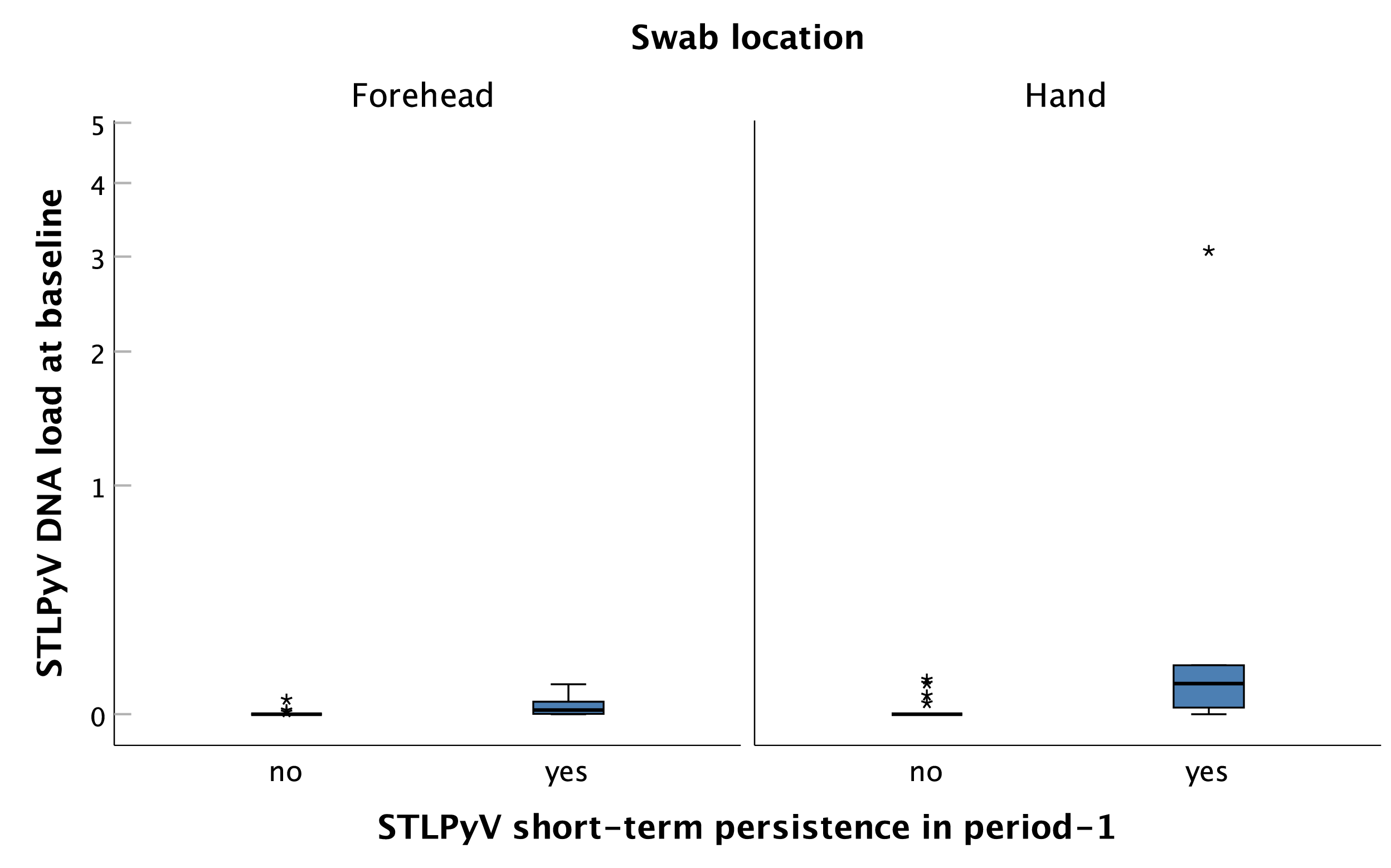  **C** |

**FIGURE S1 Baseline viral DNA loads of HPyV7, HPyV10 and STLPyV on the forehead and the hand of individuals without and with short-term persistence.** Viral DNA loads (y-axis) were expressed as viral DNA copies per betaglobin-gene copy. The left box plot shows the baseline viral DNA load distribution of individuals without short-term persistence, and the right box plot that of individuals with short-term persistence in collection period-1, respectively. Boxes represent the interquartile range with the median. Whiskers are vertical lines ending in horizontal lines at the largest and smallest observed values that are not statistical outliers, i.e. values more than three IQRs from the end of a box are labeled as extreme, denoted with an asterisk (*) and values more than 1.5 IQRs but less than 3 IQRs from the end of the box are labeled as outliers (o).

(**A)** HPyV7 DNA loads. The differences in viral DNA loads between individuals without and with short-term persistence were significant for both locations (p < 0.001 for forehead (n = 109) and hand (n = 109), respectively; independent-samples Mann-Whitney U test), (**B)** HPyV10 DNA loads. The differences in viral DNA loads between individuals without and with short-term persistence were significant for both locations (p < 0.001 for forehead (n = 109) and hand (n = 109), respectively; independent-samples Mann-Whitney U test), (**C**) STLPyV DNA loads. The differences in viral DNA loads between individuals without and with short-term persistence were significant for both locations (p < 0.001 for forehead (n = 109) and hand (n = 109), respectively; independent-samples Mann-Whitney U test).

| **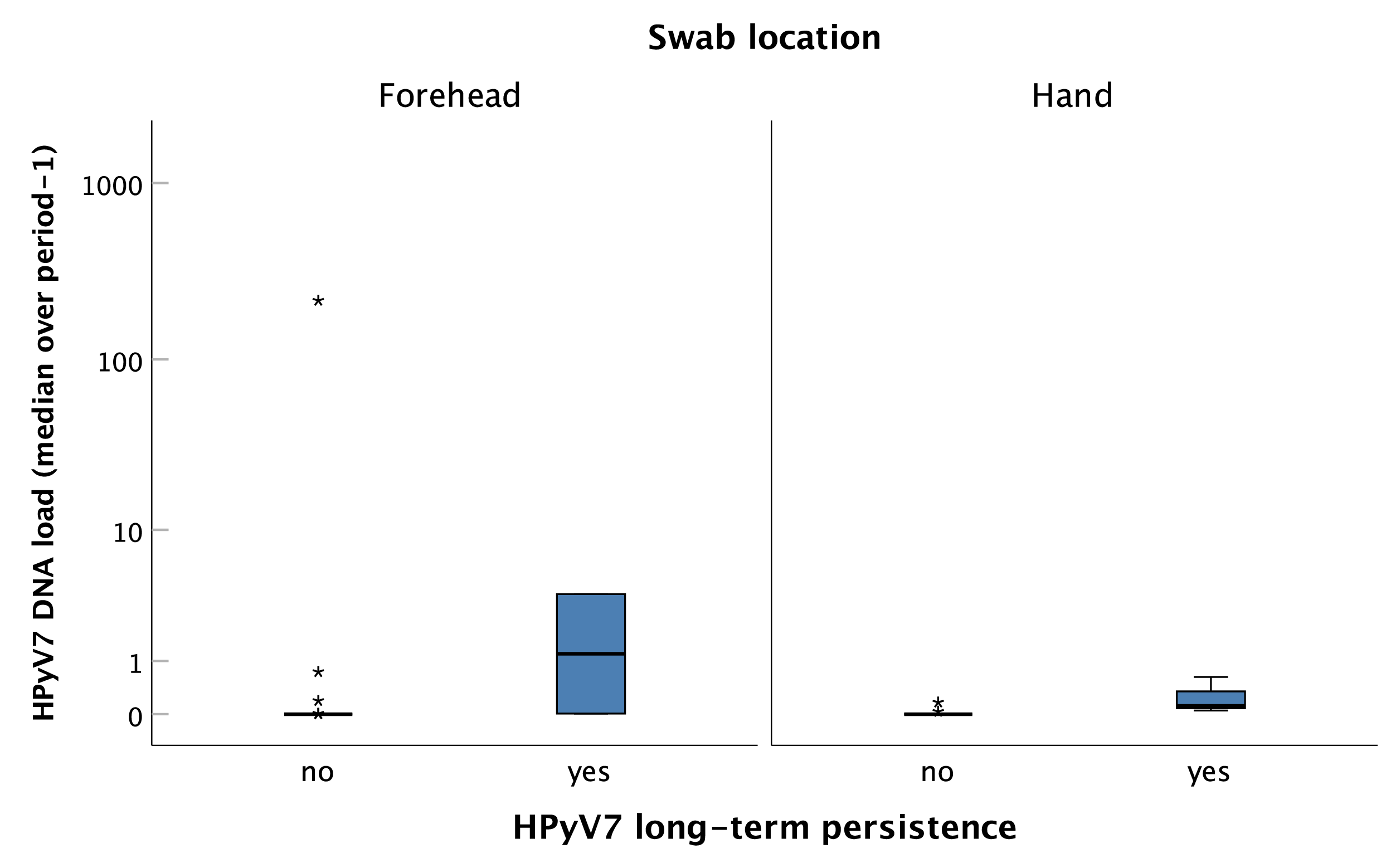** |
| --- |

**FIGURE S2** HPyV7 median viral load over period-1 on the forehead and the hand of individuals without and with long-term persistence.

The left box plot shows the distribution of the median viral load over collection period-1 of individuals without long-term persistence, and the right box plot that of individuals with long-term persistence, respectively. See legend to Figure S1 for definition of viral load and description of box plots. The differences in viral DNA loads between individuals without and with long-term persistence were significant for both locations (p = 0.011 for forehead (n = 59) and p < 0.001 for hand (n = 59); independent-samples Mann-Whitney U test).
